# The influence of epidural analgesic techniques on obstetrical outcomes

**DOI:** 10.1007/s00404-024-07591-2

**Published:** 2024-06-16

**Authors:** Christian Wiesmann, Alex Horky, Anna Hentrich, Franz Bahlmann, Frank Louwen, Ammar Al Naimi

**Affiliations:** 1 Department of Gynecology and Obstetrics, School of Medicine, Goethe University, Theodor-Stern-Kai 7, 60590 Frankfurt, Germany; 2 Department of Gynecology and Obstetrics, Buergerhospital, Nibelungenallee 37-41, 605318 Frankfurt, Germany; 3 Department of Obstetrics, Goethe-University Hospital, Theodor-Stern-Kai 7, 60590 Frankfurt, Germany

**Keywords:** Epidural analgesia, Obstetrical outcomes, Programmed intermittent epidural bolus, Patient-controlled epidural analgesia, Duration of labor

## Abstract

**Purpose:**

The aim of this study is to compare and evaluate the obstetrical differences between three techniques, including the programmed intermittent epidural bolus (PIEB), the patient-controlled epidural analgesia (PCEA), and the continuous epidural analgesia (CEA).

**Methods:**

This is a retrospective cohort study that investigates the obstetrical outcomes of 2240 patients who received EA during labor in a tertiary maternal unit over the course of 9 years (2011–2018). The only inclusion criterion was the use of epidural analgesia during childbirth and the only exclusion criteria were multiplets’ gestation. Multivariate logistic regression, Kruskal–Wallis test, and the log-rank test were utilized to compare the differences between the three EA techniques in terms of cesarean section rate, the incidence of perineal tears, the use of Oxytocin, the duration of labor, and the incidence of paresthesia.

**Results:**

Out of the 2240 included deliveries; 1084 utilized PIEB, 1086 PCEA, and 70 CEA techniques. The incidence of Cesarean section was the highest in the CEA group (45.7%) compared to PIEB (24.8%) and PCEA (24.4%) *P* < 0.001. A significantly shorter duration of labor (vaginal delivery) was observed in the PCEA group (*n*: 821, 336.7 min) compared to the PIEB group (*n*: 814, 368.8 min) *P* < 0.001. There were no statistically significant differences in the incidence of perineal tears, the need of uterotonics, and the incidence of paresthesia.

**Conclusion:**

The results of this study indicate that the PIEB and PCEA techniques are superior to the CEA technique when it comes to analgesia during childbirth. In this study, the PCEA technique seems to be the best-suited technique for childbirth, since it had a significantly shorter duration of labor than the PIEB technique.

## What does this study add to the clinical work

**Table:** 

This study examines the influence of epidural analgesic techniques on obstetrical outcomes and shows that labor duration is reduced by 32 min after PCEA compared to PIEB.	

## Introduction

Epidural analgesia (EA) is a form of regional anesthesia and is one of the neuroaxial anesthetic methods. A regional nerve block is achieved by injecting a local anesthetic with or without the addition of an opioid into the epidural space of the spinal canal [1]. It is used for post-operative, post-traumatic and for chronic pain therapy. Moreover, it is one of the most effective and safe pain management options during childbirth [2–4].

The obstetrical side-effects of EA have been widely discussed. Aside from the general side-effects of EA, prolongation of the second phase of labor and a higher incidence of vaginal instrumental deliveries are labor-specific side-effects [1, 5–7]. Recent evidence suggests no causal association between epidural labor analgesia and increased cesarean delivery rates [8]. Additionally, the American College of Obstetrics and Gynecology in their practice bulletin [9] indicated that “Randomized trials and systematic reviews including thousands of patients have shown that the initiation of epidural analgesia at any stage during labor does not increase the risk of cesarean delivery” [1, 10–12]. The interplay between oxytocin and EA in childbirth exhibits multifaceted dynamics, and hypotheses regarding a potential influence of EA on uterine myometrial activity or the causation of fetal abnormal presentation and position remain inadequately addressed in existing literature [5].

EA’s safety has steadily improved over the years through newer medications, techniques, and dosage systems [13]. Three different forms of EA application include the programmed intermittent boluses (PIEB), continuous epidural analgesia via a syringe pump (CEA), and patient-controlled epidural analgesia (PCEA). Patients with PIEB receive an automatic bolus every 60 min. They are able to expedite the administration of the next bolus 30 min after the last. This action postpones the next automatic bolus to 60 min. Patients with PCEA only receive a bolus by pressing a button and this blocks the ability to initiate another bolus for 30 min. While CEA patients receive a continuous infusion through the epidural catheter [14, 15]. Individual administrations that have been used will be discussed.

The aim of this study is to compare the three aforementioned EP procedures in term of their obstetrical outcomes with a focus on the incidence of cesarean section, perineal tears, the use of uterotonics, the frequency of paresthesia, and the duration of labor.

## Materials and methods

This is a retrospective cohort study investigating the obstetrical outcomes of 2240 patients who received EA during labor in a tertiary maternal unit, that became the largest perinatal center in Germany as of 2022, over the course of 9 years (2011–2018). There were no differences in obstetric or neonatal practices during this time period.

The study was approved by the local Ethics Committee of the University of Frankfurt (reference number 2023–1182) and patient-specific consent was not required. The only inclusion criterion was the use of epidural analgesia during childbirth and multiplet gestations were excluded.

Alongside administered neuraxial pain management techniques, diverse alternative methods were frequently employed as standalone therapies or in combination, including homeopathy, transcutaneous electrical nerve stimulation (TENS), analgesia delivered orally, transrectally, intravenously, and via nitrous oxide inhalation. The perceived inadequacy of these alternatives in achieving sufficient pain relief constituted the most prevalent reason for requesting epidural analgesia. The use of those non-neuraxial pain management techniques was not assessed within the parameters of this study.

The type of EA was the exposure of interest, and it was a categorical variable with three categories, including PIEB, PCEA, and CEA. The usual configuration of the CEA was 40 mL of 0.2% ropivacaine (2 mg/mL) with 10 µg of sufentanil delivered at a rate of 4.5 mL/h. The most frequently recorded dose for PCEA boluses was 8 mL of 0.11% ropivacaine (0.88 mg/mL) with 0.5 µg/mL sufentanil. Whereas the most commonly documented doses for PIEB boluses were an 8 mL auto bolus and a 6 mL on-demand bolus, both containing ropivacaine 0.83 mg/mL and sufentanil 0.42 µg/mL. The choice of method was driven by the prevailing standard practice at the time of implementation, and the oldest technique, i.e., CEA, eventually gave way to PCEA and subsequently PIEB, during the course of this study representing an advancement in the standard of care based on evolving evidence.

After adequate pain management and a resting period of at least 90 min, a vaginal digital examination was performed to evaluate the progress of cervical dilatation. Oxytocin infusion as augmentation was indicated in the absence of progress and persistence of optimal conditions, such as fetal position, continued satisfaction and consent of the patient, etc. Oxytocin administration was based on 3 IU oxytocin diluted in 500 mL of sodium sulfate solution, and the initial infusion rate was set at 30 mL/h with subsequent gradual increases in the absence of labor progress while maintaining adequate pain relief.

Analyzed variables for covariates and outcomes included maternal age, body mass index (BMI), parity, fetal birth weight, head circumference, arterial pH, 5-min APGAR score, the mode of delivery, cervical dilatation at time of EA application, the duration of labor post EA in minutes, the incidence of 3rd and 4th degree perineal tears, the use of oxytocin, the incidence of paresthesia, technical difficulties including the spinal or intravascular application of EA, the leak of liquor, or bone contact. All statistical analysis was conducted with Stata^®^ (ver. 16.1, Texas, USA) using Pearson’s chi-square test, univariate and multivariate logistic regression, Kruskal–Wallis test as well as the log-rank test and Kaplan–Meier curves. The cut-off point for significance was *P* value of 0.05.

## Results

The overall cesarean section rate for singleton pregnancies at the study center within the examined period was 30.2%, and half of which (14.1%) were unplanned. The prevalence of EA among all women undergoing unplanned cesarean delivery was 28.4%. Out of the 2240 included deliveries, 1084 (48.4%) utilized PIEB, 1086 (48.5%) PCEA, and 70 (3.1%) CEA techniques.

There was no statistically significant difference in terms of maternal age, parity, BMI, cervical dilatation at the time of EA, and the history of cesarean section between the three groups. The results of these comparisons are shown in Table 1.

**Table 1:** The demographic characteristics of the three cohorts

*N* (2240)	PIEB (1084) Mean ± SD Median (IQR) Frequency (%)	PCEA (1086) Mean ± SD Median (IQR) Frequency (%)	CEA (70) Mean ± SD Median (IQR) Frequency (%)	*P* value
Maternal age (years)	31.98 ± 5.32	31.86 ± 5.26	32.19 ± 5.67	0.83
Parity	1 (1–2)	1 (1–2)	1 (1–2)	0.80
BMI (kg/m^2^)	29.07 ± 5.13	29.06 ± 5.14	29.2 ± 5.34	0.87
Dilatation at epidural (centimeter)	4 (3–6)	4 (3–6)	4 (3–6)	0.75
History of cesarean section in multiparity	78 (27.4%)	81 (29.1%)	6 (42.9%)	0.43

*PIEB* programmed intermittent boluses, *CEA* continuous epidural analgesia, *PCEA* patient-controlled epidural analgesia, *BMI* body mass index, *SD* standard deviation, *IQR* interquartile range

The fetal outcomes, including weight, head circumference, arterial pH, and the APGAR scores, did not significantly differ between the three cohorts. Similarly, the technical difficulties and the incidence of paresthesia associated with EA application did not significantly differ.

There was no difference in the use of oxytocin between PIEB, PCEA, and CEA (85.1%, 84.1% and 78.8%, respectively) with 0.528 *P* value. Patients who received CEA had a significantly higher incidence of cesarean section (*N*: 32, 45.71%) compared to PIEB (*N*: 269, 24.82%) and PCEA (*N*: 265, 24.40%), but there was no statistically significant difference between PIEB and PCEA (*P* value 0.823).

**Fig. 1:**
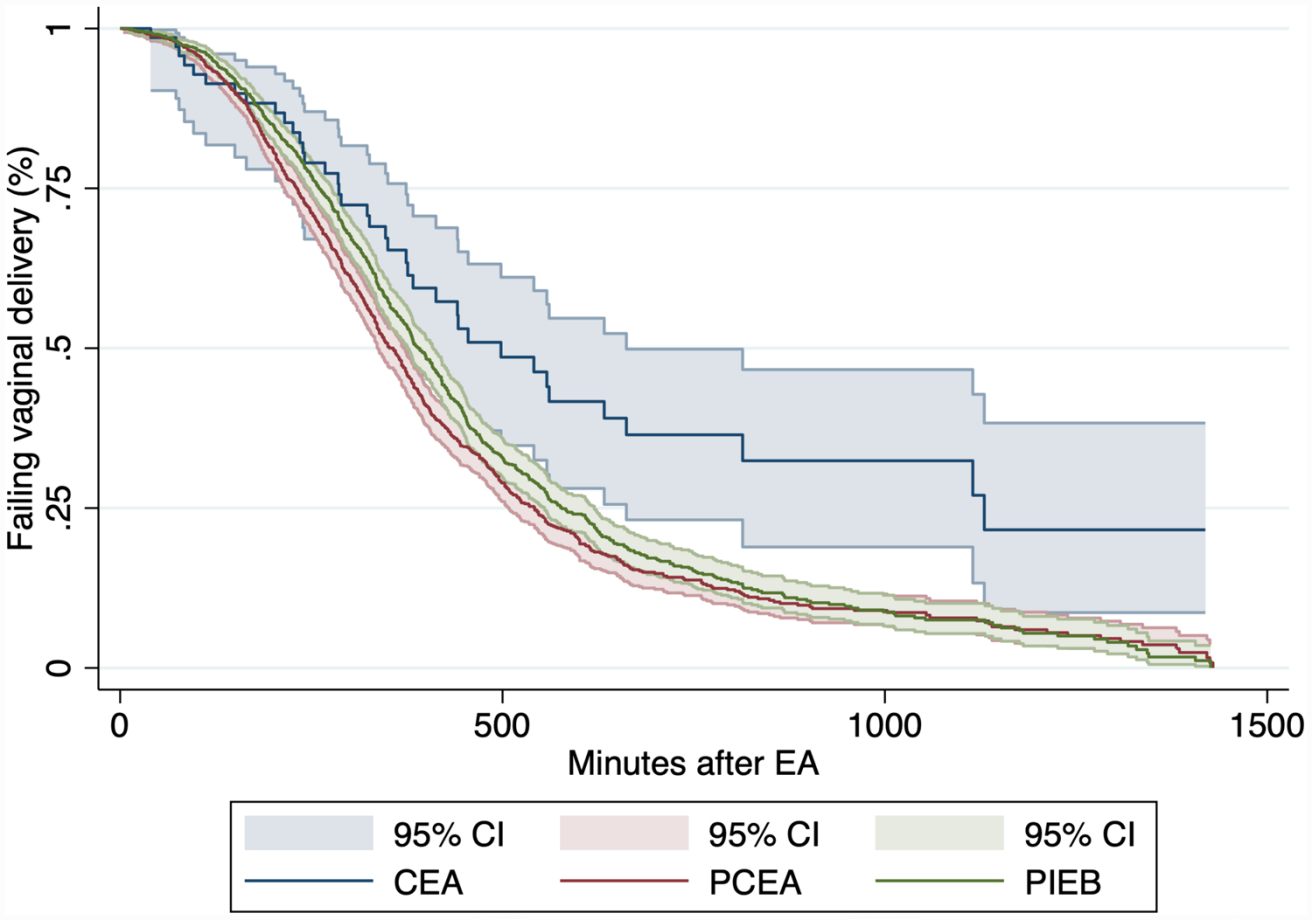
Kaplan–Meier analysis for the time to vaginal birth for the three cohorts. *PIEB* programmed intermittent boluses, *CEA* continuous epidural analgesia, *PCEA* patient-controlled epidural analgesia

Around 7% of women who achieved vaginal delivery experienced severe perineal tears regardless of the cohort (*P* value 0.88). The average duration of labor after EA is about 32 min shorter in patients who received PCEA (*n*: 821, 336.7 min) than those with PIEB (*n*: 814, 368.9 min). This corresponds to a comparative shortening of labor by about 8.7%. Women with The CEA showed the longest average delivery time of 369.2 min. The Kaplan–Meier curves for the time of labor from the application of EA to vaginal delivery are shown in Fig. 1. These differences in time to delivery were statistically significant with 0.0001 *P* value.

The obstetrical outcomes of the three cohorts are summarized in Table 2.

**Table 2:** The obstetrical outcomes for the three cohorts of the study with the corresponding *P* value of the adjusted multivariate logistic or linear regression analyses

	PIEB Mean ± SD Median (IQR) Frequency (%)	PCEA Mean ± SD Median (IQR) Frequency (%)	CEA Mean ± SD Median (IQR) Frequency (%)	*P* value
Fetal weight (g)	3437 ± 514	3465 ± 502	3507 ± 462	0.39
Head circumference (cm)	34.93 ± 1.5	34.98 ± 1.5	35.01 ± 1.4	0.73
Parasthesia	17 (1.5%)	26 (2.3%)	2 (2.8%)	0.34
Technical difficulties	65 (5.8%)	70 (6.2%)	1 (1.4%)	0.23
Severe perineal tears in vaginal	31 (7.5%)	30 (7.4%)	1 (7.1%)	0.88
Unplanned cesarean	269 (24.8%)	265 (24.4%)	32 (45.7%)	< 0.0001
Epidural to vaginal birth time (min)	392.3 ± 228.4	362.3 ± 222.9	442.1 ± 323.3	0.0001
Instrumental delivery	244 (21.8%)	194 (17.3%)	12 (16.9%)	0.22
Arterial Ph	7.24 ± 0.08	7.22 ± 0.01	7.21 ± 0.09	0.35
5’ APGAR	10 (9–10)	10 (10–10)	10 (9–10)	0.71

*PIEB* programmed intermittent boluses, *CEA* continuous epidural analgesia, *PCEA* patient-controlled epidural analgesia, *SD* standard deviation, *IQR* interquartile range

## Discussion

This study includes relatively large cohorts of women with either PCEA or PIEB under labor and a comparatively smaller cohort of women with CEA. Nevertheless, the number of observations in each cohort was large enough to produce statistically significant results where clinical inference can be achieved. The baseline demographic characteristics of the three cohorts are homogeneous, which renders them comparable and reduces the likelihood of observing confounded outcomes.

The correlation between the requirement for oxytocin augmentation and EA is widely acknowledged in academic literature [16]. Nevertheless, the rate of oxytocin augmentation in our cohorts was exceptionally high around 80% which might simply be reflective of the institutional policy. Any differences in obstetrical outcomes between the three EA cohorts should not be attributed to oxytocin augmentation due to the nondifferential distribution of its usage.

We found that CEA almost doubled the risk of cesarean section compared to PIEB and PCEA with an odds ratio about 2.55 and *P* < 0.001. The risk of cesarean section among patients who received CEA was 45.7% compared to 24.8% in the PIEB group and 24.4% in the PCEA group. Several published papers contradict this finding and report no differences in the incidence of unplanned cesarean section for CEA compared to PIEB [13]. Therefore, it is important to critically interpret our results and acknowledge that the CEA group had a limited sample size of only 70 which could affect the generalizability and the representation of the group. PIEB and PCEA, on the other hand, did not affect the risk of cesarean section which is in agreement with randomized-controlled trials that found no differences in the mode of delivery or perineal injuries between PIEB and PCEA [17].

Furthermore, we have observed that PCEA is associated with a statistically significant reduction in the duration of labor, including both the first stage with cervical dilatation and the active second stage. PCEA leads to approximately 32 min shorter duration of labor compared to PIEB with a *P* value of 0.011. We could not find explicit reports on the duration of labor for PIEB compared to PCEA in published literature, but several publications conclude that PCEA is superior to PIEB due to its association with lower drug consumption [18]. Other studies showed that PIEB leads to a shorter second stage of labor with less motor block and drug usage compared to CEA [19].

Due to the nature of the retrospective study design and the limitations of the available data, it was impossible to analyze the birth duration under the two separate stages of labor. This might introduce potential confounding factors and impact our ability to reach more specific conclusions about the duration of each stage individually. Moreover, the timing of EA application is another factor that influence the measured duration of labor. Application timing is dependent on various factors such as personnel-related practices, and thus represents an uncontrolled covariant. However, since there was no significant difference between the compared groups regarding cervical dilatation at the time of EA application, it is unlikely that the timing of EA significantly affected the measured duration of birth and, consequently, the study results.

In summary, this study shows that PIEB and PCEA are more suitable than CEA as epidural analgesic methods for obstetrical use. This is reflected in the gradual decrease in obstetrical CEAs throughout the years as the number of studies showing the superiority of PIEB and PCEA increases [20]. Finally, PCEA seems to be the most advantageous epidural procedure because of a shorter birth duration than PIEB knowing that both techniques provide comparable pain management and patient’s satisfaction [17].

## Data Availability

Not applicable.
